# RNAi-mediated silencing of *Trichinella spiralis* glutaminase results in reduced muscle larval infectivity

**DOI:** 10.1186/s13567-021-00921-1

**Published:** 2021-03-25

**Authors:** Yuan Gao, Xiaoqing Meng, Xiao Yang, Shi Meng, Caixia Han, Xiaoyun Li, Shuang Wang, Wei Li, Mingxin Song

**Affiliations:** Heilongjiang Key Laboratory for Zoonosis, College of Veterinary Medicine, Northeast Agricultural University, 600 Changjiang Street, Harbin, 150030 China

**Keywords:** *Trichinella spiralis*, Acid resistance, RNAi, Glutaminase

## Abstract

*Trichinella spiralis* is an important foodborne parasitic nematode distributed worldwide that infects humans and animals. Glutaminase (GLS) is an important gene in the glutamine-dependent acid resistance (AR) system; however, its role in *T. spiralis* muscle larvae (ML) remains unclear. The present study aimed to characterize *T. spiralis* GLS (TsGLS) and assess its function in *T. spiralis* ML AR both in vitro and in vivo using RNA interference. The results indicated that native TsGLS (72 kDa) was recognized by anti-rTsGLS serum at the muscle larvae stage; moreover, an immunofluorescence assay confirmed that TsGLS was located in the epidermis of ML. After silencing the TsGLS gene, the relative expression of TsGLS mRNA and the survival rate of *T. spiralis* ML were reduced by 60.11% and 16.55%, respectively, compared to those in the PBS and control groups. In vivo AR assays revealed that the worm numbers at 7 and 35 days post-infection (dpi) decreased by 61.64% and 66.71%, respectively, compared to those in the PBS group. The relative expression of TsGLS mRNA in F_1_ generation *T. spiralis* ML was reduced by 42.52%, compared to that in the PBS group. To the best of our knowledge, this is the first study to report the presence of the glutamine-dependent AR system in *T. spiralis*. Our results indicate that TsGLS plays a crucial role in the *T. spiralis* AR system; thus, it could be used as a potential candidate target molecule for producing vaccines against *T. spiralis* infection.

**Supplementary Information:**

The online version contains supplementary material available at 10.1186/s13567-021-00921-1.

## Introduction

*Trichinella spiralis*, a causative agent of trichinellosis, is an important foodborne parasitic nematode which is distributed worldwide [1]. Trichinellosis is an emerging/re-emerging disease [1, 2]; *T. spiralis* can infect various hosts, including humans. Infection is caused by ingesting raw or semi-raw meat contaminated by infective *T. spiralis* larvae [3]. Trichinellosis poses public health concerns, and causes economic issues in porcine animal production and food safety [4]. Drug treatment is not effective in preventing recurring infection of *T. spiralis* due to lack of specific clinical symptoms or signs for its diagnosis*.* Thus, the development of therapeutic methods and anti-*Trichinella* vaccines is of utmost importance.

The acid resistance (AR) mechanism exists in several foodborne pathogenic bacteria, including *Escherichia coli*, and allows their survival in various acidic conditions [5]. Five distinct amino acid-dependent AR systems have been characterized in *E. coli*, comprising the glutamic acid-, arginine-, lysine-, ornithine-, and glutamine (Gln)-dependent AR systems [6, 7]. The Gln-dependent AR system is considered highly effective, and relies on L-Gln, one of the most abundant food-borne free amino acids. Upon uptake into *E. coli*, Gln is converted into L-glutamate (Glu) by acid-activated glutaminase (GLS), with the concomitant release of gaseous ammonia. The free ammonia neutralizes protons, resulting in the elevation of the intracellular pH under acidic conditions [7]. The AR system relies on GLS and is activated by acidic pHs; Gln is also an essential component of this AR, enabling its activity in acidic environments.

The life cycle of *T. spiralis* indicates that the larvae are retained in the acidic environment of the stomach [8]. Thus, we speculated that AR systems may exist in *T. spiralis* muscle larvae (ML). Importantly, one study reported a markedly high content of Gln in *T. spiralis* larvae [9]. In recent years, RNA interference (RNAi) technology has been used as an effective tool to study the function of genes in various fields, including parasitology. For example, SjVasa3 gene silencing in *Schistosoma japonicum* using RNAi technology can significantly reduce egg production [10]. Moreover, nudix hydrolase-specific small interfering RNA (siRNA) was successfully used to inhibit the expression of nudix hydrolase in *T. spiralis*, reducing the invasiveness of *T. spiralis* larvae and improving the rate of host survival [11].

In the present study, TsGLS was selected and expressed to demonstrate the existence of AR systems in *T. spiralis* ML. Furthermore, we investigated the functions of TsGLS in *T. spiralis* AR systems, and TsGLS-specific siRNAs were designed to silence the expression of TsGLS in *T. spiralis* larvae to elucidate the functions of this gene.

## Materials and methods

### Parasites and experimental animal models

The *T. spiralis* strain (*T. spiralis* ISS533) was obtained from Chinese native black pigs in a commercial slaughter house in Heilongjiang Province, China, and was preserved using Kunming mice in the Department of Parasitology, College of Veterinary Medicine, Northeast Agricultural University. Female BALB/c mice (6–8 weeks, 18–22 g) and New Zealand white rabbits (about 2 kg) were purchased from the Experimental Animal Center at Harbin Medical University. The bodies of mice infected with *T. spiralis* were digested using artificial gastric juice containing 1% pepsin and 1% concentrated hydrochloric acid. Thereafter, *T. spiralis* ML samples were collected using the modified Baermann method post digestion at 37 ℃ for 3 h [12]. The experiments conducted in this study were approved by the Animal Ethics Committee of Harbin Medical University and were performed in accordance with animal ethics guidelines and approved protocols (Animal Ethics Committee approval number SYXK [Hei] 2016-007).

### Recombinant protein expression and polyclonal antibody (Ab) production

TsGLS gene was amplified via PCR using specific primers with SacI and HindIII restriction enzyme sites. The primers were designed by the corresponding sequences available in GenBank (GenBank accession no. XM_003375164). The purified PCR product was cloned into the pMD18-T vector, and then subsequently subcloned into the pET-32a. Thereafter, the recombinant plasmid carrying the TsGLS gene was transformed into Rosetta (DE3) (TransGen Biotech, Beijing, China) and expressed under isopropyl β-d-1-thiogalactopyranoside induction. Moreover, the recombinant TsGLS (rTsGLS) was purified using freeze–thaw cycles. The purified rTsGLS was identified by sodium dodecyl sulfate polyacrylamide gel electrophoresis (SDS-PAGE).

New Zealand white rabbits were used to produce anti-rTsGLS serum. First, the rabbits were immunized subcutaneously with rTsGLS emulsified with complete Freund’s adjuvant. Thereafter, two booster immunizations were carried out at 3 and 28 days after first immunization by injecting the rabbits with the same amount of rTsGLS emulsified with incomplete Freund’s adjuvant. Blood samples were collected from the immunized rabbits at Day 7 after the last immunization, and the sera were obtained. Furthermore, anti-rTsGLS serum was stored at – 80 ℃ for subsequent studies. The polyclonal Ab titer of rTsGLS was measured by ELISA.

### Western blot analysis

First, recombinant protein and crude protein samples from ML were separated via SDS-PAGE on a 12% acrylamide separation gel and subsequently transferred onto a polyvinylidene difluoride membrane. Second, the membranes were blocked with 5% (w/v) skim milk powder and incubated with anti-rTsGLS serum (1:1000). GAPDH expression was detected as a quantitative protein control using rabbit anti-GAPDH (1:10 000; Proteintech Group, Chicago, USA). Thereafter, horseradish peroxidase-labeled goat anti-rabbit IgG (H + L) (1:10 000, Proteintech Group) was used as the secondary antibody. Eventually, the reaction was detected using the GeneSys detection system (Syngene, Cambridge, UK) and analyzed using Image J [13].

### Quantitative real-time PCR (qRT-PCR)

The total RNA of *T. spiralis* ML was extracted using TRIzol reagent (Invitrogen, Carlsbad, USA). Specificity primers were designed according to the TsGLS (XM _003375164) available in GenBank, as follows: TsGLS-F: ATCCGAACAAGGGCAACTG, TsGLS-R: CGGCACTGATACCAAACCAT. The GAPDH gene of *T. spiralis* ML was used as a housekeeping gene, as follows: TsGAPDH-F: TGGCTTAGCTCCGTTGG, TsGAPDH-R: TTTGGGTTGCCGTTGTA. qRT-PCR was performed in triplicate using the SYBR Premix Ex Taq™ II (Perfect Real Time) (Takara, Dalian, China) and ABI 7500 (Life Tech [Applied BioSystems], Waltham, USA) to evaluate the transcription levels of the target gene.

### Immunofluorescence assay (IFA)

The diaphragms of mice infected with *T. spiralis* ML were used for IFA [14]. Eight micrometer-thick sections were prepared using a microtome. After blocking with normal goat serum, the sections were incubated with anti-rTsGLS serum (1:1000 dilutions) at 37 °C for 1 h. After washing with phosphate-buffered saline (PBS), these sections were incubated with fluorescein-labeled goat anti-rabbit IgG (1:1000 dilution). We observed the sections under a fluorescence microscope.

### Effect of acidic conditions on ***T. spiralis*** ML GLS

To test the effect of acidic conditions on *T. spiralis* ML GLS mRNA, *T. spiralis* ML samples (about 1000 worms) were cultured at pH 2.0, 4.0, 6.6, and 9.0 at 37 °C and 5% CO_2_ for 0.5, 1, and 3 h, each. To evaluate the effect of pepsin on TsGLS, each group of *T. spiralis* ML was cultured in artificial pepsin (pH = 2.5), saline (pH = 2.5), and saline (pH = 6.6) for 3 h at 37 °C and 5% CO_2_. Thereafter, qRT-PCR and IFA were performed to determine the transcription and expression levels of TsGLS mRNA.

### Preparation of siRNAs and soaking of *T. spiralis* ML with siRNA

Full-length cDNA encoding TsGLS (XM_003375164) was used to design siRNA sequences with the Invitrogen BLOCK-iT RNAi Designer online service. TsGLS-specific siRNA oligos (Stealth™ RNAi duplexes) were chemically synthesized by GenePharma (Shanghai, China). The sequences of the three specific siRNA (siRNA-419, siRNA-881, siRNA-1429) oligonucleotides used in this study to prevent off-target effects and the corresponding stealth control siRNA are shown in Additional file 1. Fluorescent protein-labeled control siRNA was used to monitor the transfection efficiency.

Five groups were set: siRNA-419, siRNA-881, siRNA-1429, control siRNA, and PBS. Each group (comprising approximately 5000 worms) was cultured in 500 μL of RPMI 1640 with 2 μM siRNA and 2 μL of liposome for 6 h and 12 h, respectively. Thereafter, qRT-PCR was performed to analyze the TsGLS mRNA transcription in siRNA-treated ML as mentioned above, and the TsGLS protein expression was evaluated by Western blot analysis.

To optimize the experimental conditions, *T. spiralis* ML were soaked in 1, 2, and 4 μM siRNA-881 for 12 h and cultivated in RPMI 1640 medium at 37 °C under 5% CO_2_ conditions for 0, 1, 3, 5, and 7 days. TsGLS gene mRNA and protein expression levels were analyzed via qRT-PCR and Western blot, respectively.

### Effects of acidic conditions on the survival rate and mRNA transcription of siRNA-881-treated *T. spiralis* ML

The siRNA-881-treated *T. spiralis* ML samples were cultured at pHs of 2.5, 4.0, 6.6, and 9.0 RPMI 1640 at 37 °C for 0.5, 1, and 2 h. The survival rate of *T. spiralis* ML was calculated based on its viability. Inactive and straight (“C”-shaped) parasites were counted as dead. The live larvae were active and wriggled [11]. The death rate was calculated as follows: Death rate = [Total number of larvae – Number of dead larvae]/Total number of larvae × 100%. Moreover, qRT-PCR was performed to analyze TsGLS mRNA transcription in siRNA-treated ML, as mentioned previously.

### Effects of RNAi-881 on the activity of TsGLS

Crude protein extracts were obtained from *T. spiralis* ML treated with siRNA to test the effects of RNAi-881 on the enzymatic activity of TsGLS. The pH of the medium was measured using a pH meter when siRNA-881-treated *T. spiralis* ML were cultured at pHs of 2.5, 4.0, 6.6, and 9.0 (in RPMI 1640) at 37 °C for 0.5, 1, and 2 h.

### Effects of Gln deficiency on the AR of siRNA-881-treated *T. spiralis* ML

The *T. spiralis* ML were treated with siRNA-881 and small-molecule inhibitor bis-2-(5-phenylacetamido-1,3,4-thiadiazol-2-yl) ethyl sulfide (BPTES) [15]. Thereafter, it was cultured in 10 mM Gln medium (control group at a pH of 2.5) for 2 h. The variation of Gln and GLS expression in *T. spiralis* ML in the acidic microenvironment was detected via qRT-PCR. The survival rate of *T. spiralis* ML was calculated as follows: Survival rate = [Total number of larvae – Number of deaths]/Total number of larvae × 100%.

### Assessment of the effects of RNAi on the AR of *T. spiralis* ML via in vivo assays

Thirty 6-week-old BALB/c mice were divided into three groups (10 mice in each) and orally inoculated with 300 siRNA-811-, control siRNA-, or PBS-treated *T. spiralis* ML. The adult worms were collected from the intestine of 15 infected mice at 7 days post-infection (dpi) and the worm reduction was enumerated after they were incubated individually in RPMI-1640 medium for 3 h.

ML were collected from 15 infected mice at 35 dpi by artificial digestion, as described in a previous study [16]. Hematoxylin–Eosin staining was used to compare the difference in collagen capsule thickness, as well as the aggregation of eosinophils and basophils in the nurse cell at 35 dpi. The worm reduction was calculated using the following formula: Worm reduction = (Number of worms in PBS group – Number of worms in siRNA-881 group)/Number of worms in PBS × 100%. The ML samples were cultured at a pH of 2.5 for 2 h to detect the TsGLS gene transcription level.

### Statistical analysis

Statistical analysis was performed using SPSS 12.0 software. Comparison among groups was analyzed by one-way ANOVA. The results were expressed as mean ± standard deviation (SE). Graphs were drawn using GraphPad Prism 5.0 software.

## Results

### Molecular cloning, expression, and location of the cDNA encoding a 72 kDa TsGLS

**Figure 1:**
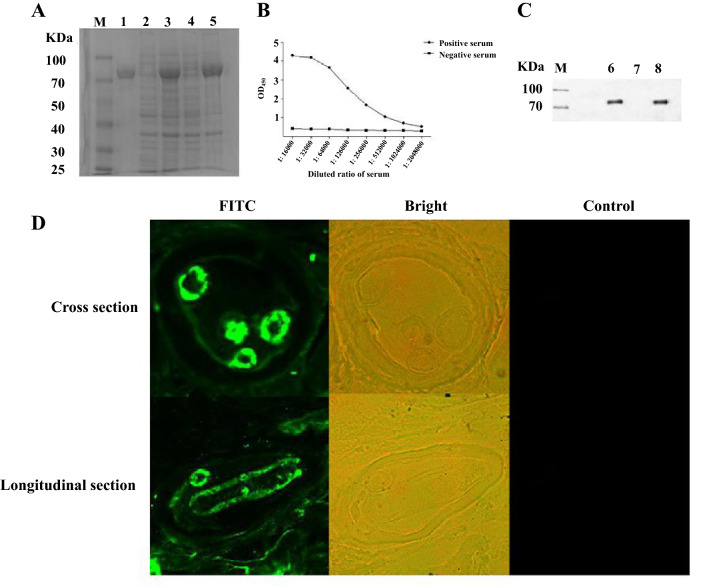
**Molecular characterization of TsGLS.** **A** SDS-PAGE analysis of TsGLS. M: protein molecular weight marker; 1: purified product; 2: pET-32a-TsGLS without induction; 3: pET-32a-TsGLS induced for 6 h; 4: supernatant sample; 5: sediment sample; **B** analysis of polyclonal Ab titer; **C** Western blot analysis, M: protein molecular weight marker; 6: rTsGLS; 7: negative control; 8: crude protein; and **D** immunofluorescence localization analysis of TsGLS.

The 1665-bp sequence of TsGLS (without a signal peptide) was amplified; it encoded 554 amino acids. After being induced with 0.5 mM IPTG, rTsGLS produced a protein band of approximately 72 kDa, as examined via SDS-PAGE, and the molecular weight of the TsGLS protein was compatible with its predicted size (Figure 1A). Moreover, ELISA revealed that the anti-rTsGLS sera strongly reacted with the rTsGLS and the polyclonal Ab titer was 1.024 × 10^6^ (Figure 1B). The rTsGLS protein and crude protein in ML were identified using anti-rTsGLS serum via Western blot analysis, wherein all specific bands were obtained, indicating that both antigens had better reactogenicity (Figure 1C). The IFA results confirmed the expression of TsGLS at the ML stage of *T. spiralis*. Furthermore, immunofluorescence staining of the epidermis of the ML was observed (Figure 1D).

### Effects of acidic pH on *T. spiralis* ML GLS

**Figure 2:**
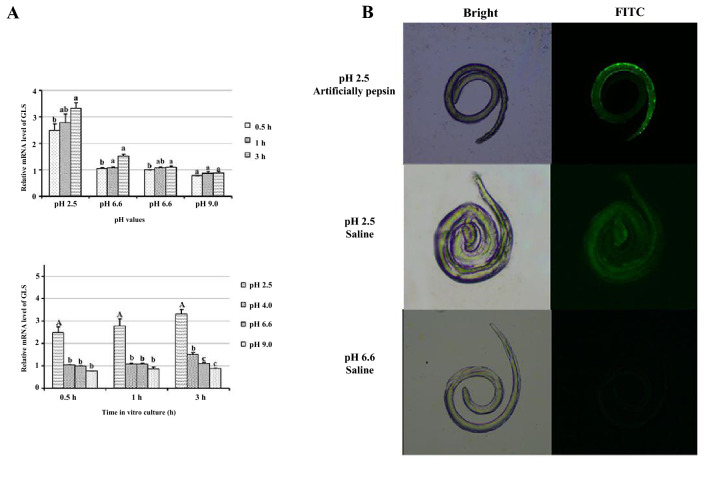
**Effect of acidic medium on TsGLS mRNA expression levels.** **A** Relative expression of TsGLS under different cultivation times and pH values, *P* < 0.01; a, b, c: *P* < 0.05; and **B** immunofluorescence signal analysis.

To test the effect of acidity on GLS in ML, *T. spiralis* ML samples were cultured in media at different pHs for various periods of time. The relative expression of TsGLS mRNA after culture at pH 2.5 for 3 h was higher than that observed following culture at other pHs (2.0, 4.0, 6.6 and 9.0) and for other culture times (0.5, 1, and 3 h) (*P* < 0.01) (Figure 2A).

When we cultured *T. spiralis* ML in a medium containing both artificial pepsin and saline (pH 2.5), the relative expression of TsGLS mRNA in this medium was higher than that at pH 6.6 (*P* < 0.01). Minimal differences were observed between artificial pepsin and saline at pH 2.5 (*P* > 0.05). The IFA results indicated that immunofluorescence staining of ML epidermis was observed when *T. spiralis* ML were cultured at pH 2.5 (Figure 2B).

### Delivery of siRNA to *T. spiralis* ML

Twelve hours after soaking with FAM-labeled control siRNA, the treated larvae displayed fluorescence staining in *T. spiralis* ML under a fluorescent microscope, however the untreated larvae did not display fluorescence staining (Additional file 2A), thereby demonstrating that siRNA can be efficiently delivered into *T. spiralis* ML by soaking.

*T. spiralis* ML samples were soaked with 2 μM siRNA-419, siRNA-881, and siRNA-1429 for 3 days. The transcription level of TsGLS mRNA in the treated larvae decreased significantly compared with that of larvae treated with control siRNA or untreated larvae (*P* < 0.01). The relative transcription levels of TsGLS in the larvae treated with siRNA-419, siRNA-881, and siRNA-1429 was 49.99%, 72.82%, and 60.69% of the levels in untreated larvae, respectively, as detected by qRT-PCR. The expression levels of the TsGLS protein were significantly reduced in larvae treated with siRNA-419, siRNA-881, and siRNA-1429, compared with those in larvae treated with control siRNA or the untreated larvae. Furthermore, compared with untreated larvae, the expression levels of the TsGLS protein were inhibited by 92.3%, 64.48%, and 44.56% when larvae were soaked with 2 μM of siRNA-419, siRNA-881, and siRNA-1429, respectively (*P* < 0.05). The control siRNA did not reduce the TsGLS gene transcription and expression levels (*P* > 0.05) (Additional file 2B).

The relative transcription levels of TsGLS in the larvae treated with 1, 2, and 4 μM of siRNA-881 for 3 days was 45.76%, 64.19%, and 71.9% lower than those in the untreated larvae, respectively; moreover, the transcription levels of TsGLS in the larvae treated with aforementioned three concentrations of siRNA-881 were significantly lower than those of the untreated larvae (*P* < 0.001). The expression levels of the TsGLS protein in larvae were inhibited by 45.72%, 63.71%, and 76.78% in the 1, 2, and 4 μM of siRNA-881 groups, respectively, compared to that in the PBS group (*P* < 0.05). After being soaked with 2 μM of siRNA-881 for 1, 3, 5, and 7 days, the relative transcription level of TsGLS in the larvae was decreased by 62.91%, 82.29%, 62.93%, and 28.02% (*P* < 0.001), respectively. The control siRNA did not reduce the TsGLS gene transcription and expression levels significantly (*P* > 0.05) (Additional file 3).

### Effect of TsGLS gene silencing on larval survival

After 2 μM TsGLS siRNA-881 was delivered into *T. spiralis* ML for 3 days, the relative expression levels of TsGLS mRNA and protein were reduced by 73.13% and 55.69%, respectively, compared with those in the PBS and control groups (*P* < 0.001). The control siRNA did not significantly reduce the TsGLS gene transcription and expression levels (*P* > 0.05). The IFA results indicated that the immunofluorescence staining intensity of the siRNA-881 treatment group (0.02533 ± 0.009366) was significantly weaker than that of the PBS group (0.06484 ± 0.005154) (*P* < 0.05); however, no difference was observed between the control siRNA (0.06687 ± 0.006897) and PBS groups (*P* > 0.05). To detect the effects of the siRNA on *T. spiralis* ML, the survival of ML was calculated; no significant difference was observed between the survival of *T. spiralis* ML in the siRNA-881 treatment group and that in the control siRNA and the PBS groups (*P* > 0.05) (Additional files 4 and 5).

### In vitro AR assays after silencing the TsGLS gene

**Figure 3:**
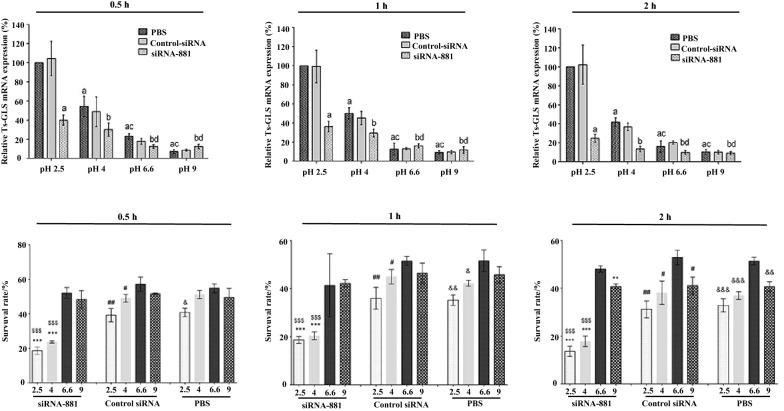
**Survival rate and relative transcription levels of TsGLS ML after treatment with siRNA-881 under different pH values and for different culture times**. All assays were performed in triplicates.

To detect the effects of siRNA-881 on *T. spiralis* ML, the survival rate of *T. spiralis* ML was calculated. The result indicated that after culturing siRNA-881-treated larvae at a pH of 2.5 for 1 h, the survival rate of *T. spiralis* ML reduced by 16.55%, compared with that of the PBS group (Figure 3).

We detected the effect on the TsGLS mRNA after silencing the TsGLS gene in acidic medium, indicating that the expression level of TsGLS mRNA increased with the decrease in the pH value after the same culture time. After TsGLS siRNA-881-treated *T. spiralis* ML samples were cultured at a pH of 2.5 for 0.5 h, the relative expression of TsGLS mRNA was reduced by 60.11% compared with that of the PBS and control groups (*P* < 0.001) (Figure 3).

### Effects of RNAi on the GLS activity of *T. spiralis* ML in acidic condition

**Figure 4:**
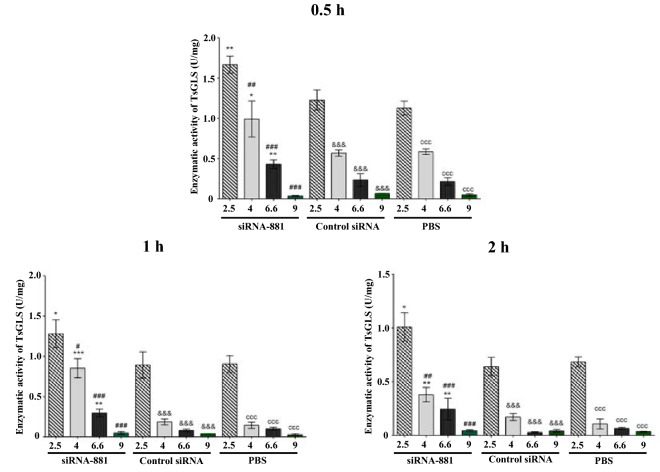
**Enzymatic activity assay in acidic medium by spectrophotometry.** *P < 0.05; **P < 0.01; **P < 0.001.

After silencing the GLS gene in *T. spiralis* ML in acidic media, the activity of GLS was found to be the highest at pH 2.5 and the lowest at pH 9.0. After TsGLS siRNA-881-treated *T. spiralis* ML were cultured at a pH of 2.5, the GLS activity at pH 2.5 was significantly higher than that in the PBS and control groups (*P* < 0.01) (Figure 4).

### Effect of RNAi on the culture medium of ML after TsGLS gene silencing

After culturing siRNA-881-treated *T. spiralis* ML in media with pHs of 2.5, 4, 6.6, and 9 for 0.5, 1, and 2 h, we found that the pH values of the culture medium with the initial pH of 2.5 in the siRNA-881 group differed slightly, compared to those in the other groups. After culturing siRNA-881-treated *T. spiralis* ML at pH 2.5 for 2 h, the pH in the siRNA-881 group was lower than that in the PBS and control groups (*P* < 0.001) (Additional file 6).

### Effects of Gln deficiency on TsGLS mRNA expression in siRNA-881-treated *T. spiralis* ML

**Figure 5:**
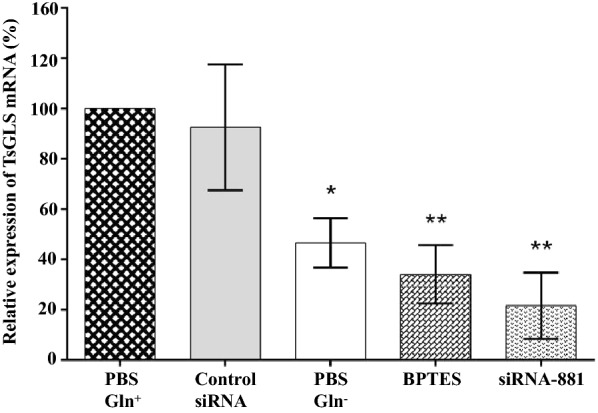
**Effects of Gln deficiency on TsGLS mRNA expression in siRNA-881-treated** *Trichinella spiralis* ML AR.*P < 0.05; **P < 0.01; **P < 0.001.

To detect the effects of Gln on *T. spiralis* ML under acid conditions, we cultured the ML under both Gln-deficient and Gln-rich conditions. The results indicated that the relative TsGLS mRNA expression levels in the PBS (Gln^–^), BPTES, and siRNA-881-treated groups were reduced by 53.48% (*P* < 0.05), 66.63% (*P* < 0.01), and 79.07% (*P* < 0.01), respectively (Figure 5).

### Assessment of the effects of RNAi on the AR of *T. spiralis* ML using in vivo assays

**Figure 6:**
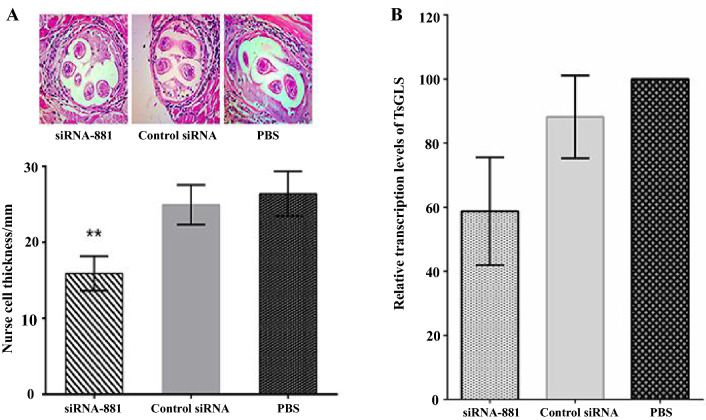
**In vivo AR assays.** **A** Hematoxylin–Eosin staining for collagen capsule thickness measurements. **B** Relative transcription levels of TsGLS in F_1_ generation siRNA-881-treated ML.

The reductions in worm numbers in siRNA-881 group at 7 and 35 dpi were 61.64% and 66.71% than that of the PBS group (Table 1). The collagen capsule of worms in the siRNA-881 group (15.90 ± 1.301) was 39.78% thinner than that of worms in the PBS (26.40 ± 1.697) and control siRNA (24.94 ± 1.511) groups (*P* < 0.01). No difference was observed between the collagen capsule thickness of worms in the control siRNA and PBS groups (*P* > 0.05) (Figure 6A). The relative expression of TsGLS mRNA in F_1_ generation *T. spiralis* ML was reduced by 42.52% compared to that in the PBS group (*P* < 0.001) (Figure 6B).

**Table 1:** **Reduction rates of siRNA-881-treated adult and larvae worms in BALB/c mice**

Group	Adult worms recovered (mean ± SE)	Reduction (%)	Larvae recovered (mean ± SE)	Reduction (%)
siRNA-881	45.80 ± 6.54	61.64	8211 ± 1356	66.71
Control siRNA	110.40 ± 11.60		23 258 ± 961.10	
PBS	119.40 ± 12.02		24 668 ± 1231	

All assays were performed in quintuplicates, and data are presented as the mean ± SE.

## Discussion

*T. spiralis* is a foodborne parasite that adapts to acid stress, which is a crucial factor for its transmission to animals. A comprehensive understanding of *T. spiralis* AR is important for the prevention and clinical treatment of *T. spiralis* infections. The genomic sequence of *T. spiralis* comprises 15 808 protein-coding genes; however, only partially functional genes have been characterized [17]. GLS plays a pivotal role in the *E. coli* AR system [7]. Till date, the biological characteristics and roles of TsGLS have not been clarified.

RNAi has been widely used in molecular biology research as an important method to study the gene functions of genes in various organisms, including a single-celled protozoon [18], multicellular fluke [19], and nematode [11]. *Nippostrongylus brasiliensis* was the first parasitic nematode in which RNAi was successfully used to suppress secreted acetylcholinesterase expression [20]. The technique can effectively evaluate the function of genes in *T. spiralis*. Previous studies have demonstrated that silencing some *T. spiralis* genes with RNAi could impair worm viability or inhibit their development, reproductive capacity, and infectivity [11, 21–23]. Silencing *T. spiralis* SPI expression indicated that this gene was crucial during the process of *T. spiralis* larval invasion and survival in the host [24]. Therefore, RNAi was used to investigate the function of TsGLS in *T. spiralis* in the present study.

In this study, a *T. spiralis* GLS gene encoding a 72-kDa protein was expressed successfully, suggesting that an AR system like that in *E. coli* might exist in *T. spiralis.* AR in bacteria is defined as the phenomenon whereby the survival rate of bacteria exceeds or equals 10% after exposure to pH < 2.5 for 2 h [25]. Moreover, culturing *T. spiralis* ML at a pH of 2.5 for 3 h resulted in a remarkably high TsGLS gene expression, which indicated that the expression of the TsGLS gene was upregulated in the acidic medium, and the survival rate of *T. spiralis* ML reduced by 16.55% after culturing the siRNA-881-treated larvae at a pH of 2.5 for 1 h. Therefore, the results indicated that AR system was existing in *T. spiralis.*

Gln could be generally important for AR in bacteria. Adding Gln allows the maintenance of a high survival rate when *E. coli* is cultured in an extremely acidic medium (pH 2.5) [5, 7]. Here, similarly, Gln deficiency affected the relative expression of TsGLS mRNA in siRNA-881-treated *T. spiralis* ML. Mammalian gastric juice pH ranges from 1.5 to 3.0 [6]. GLS could be activated under acidic conditions at pH 6.0 [7, 26]. After silencing the TsGLS gene in *T. spiralis* ML in acidic media, we found that the maximum enzymatic activity of TsGLS was higher at pH 2.5 than at pHs 4, 6.6, and 9.0, suggesting that TsGLS might be beneficial to *T. spiralis* survival in acidic environment. The highest enzymatic activity of GLS in the *E. coli* AR system was observed at pH 4 [7], which was inconsistent with the results of the present study. This difference in the maximum GLS activities might be because the GLS in each case originated from different species. Thus, elucidating the effects of the TsGLS gene on the *T. spiralis* AR requires further investigation. The composition of the mammalian gastric juice is complex; it mainly comprises hydrochloric acid and pepsin. In the present study, no remarkable differences were observed in the relative expression of TsGLS mRNA in the presence of artificial pepsin and saline at pH 2.5, suggesting that the enzymatic activity of TsGLS was not clearly inhibited by pepsin; moreover the acidic medium notably affected the TsGLS mRNA expression. *T. spiralis* ingests external nutrients by forming a capsule in the host to protect against damage from external inflammatory cells [27]. Our results revealed that the collagen capsule thickness in the siRNA-881 T*. spiralis* ML group and the relative expression of TsGLS mRNA of F_1_ generation *T. spiralis* ML were reduced by 39.78% and 42.52%, respectively, which indicated that the silencing of TsGLS might have affected the infectivity of F_1_ generation larvae and their survival in the host.

In conclusion, TsGLS was highly expressed in *T. spiralis* ML; it was mainly located in the epidermis. Silencing of the TsGLS gene by RNAi markedly reduced the TsGLS mRNA and protein expression levels, which caused the inhibition of the TsGLS activities and reduced survival of the ML. To the best of our knowledge, this is the first study using RNAi to reveal the presence of a Gln-dependent AR system in *T. spiralis.* Our results indicated that TsGLS plays an important role in *T. spiralis* AR and could be a candidate target molecule used to produce vaccines against *T. spiralis* infection.

## Supplementary Information

**Additional file 1.** **siRNA sequences for TsGLS used in this study.**

**Additional file 2.** **FAM-labeled control siRNA delivery to *Trichinella spiralis*** **ML.** A: uptake of FAM-labeled siRNA into larvae at 12 h after soaking under a fluorescent microscope. No fluorescence was observed in the untreated larvae; B: relative transcription and expression levels of TsGLS mRNA and protein in *T. spiralis* ML 3 days after being soaked with different siRNAs. Western blot with specific antibodies showing the specific inhibition of TsGLS protein expression in extracts of *T. spiralis* larvae induced by siRNAs. Statistically significant differences with *P* < 0.05, *P* < 0.01 and *P* < 0.001 are indicated by *, **, and ***, respectively.

**Additional file 3. Optimization of experimental conditions for siRNA interference. ** A: relative transcription levels of TsGLS in larvae 3 days after being soaked with different concentrations of siRNA-881; B: expression levels of the TsGLS protein in larvae 3 days after being soaked with different concentrations of siRNA-881; C: relative transcription levels of TsGLS in larvae at 1, 3, 5, and 7 days after being electroporated with 2 μM of siRNA-881. All the assays were performed in triplicates, and statistically significant differences with *P* < 0.05 and *P* < 0.001 are indicated by * and ***, respectively.

**Additional file 4. Survival rate after siRNA treatment of ML.**

**Additional file 5. TsGLS effect on larvae after silencing the TsGLS gene. ** A: relative transcription levels of TsGLS in larvae after being soaked with siRNA-881; B: expression levels of the TsGLS protein in larvae after being soaked with siRNA-881; C: immunofluorescence signal analysis and average optical density after being soaked with siRNA-881. *: *P* < 0.05; **: *P* < 0.01; **: *P* < 0.001.

**Additional file 6. RNAi effect on the culture medium of siRNA-881-treated muscle larvae under different pH values and culture times.**

## Data Availability

The datasets used or analyzed during the present study are available from the corresponding author on reasonable request.

## Abbreviations

*T. spiralis*: *Trichinella spiralis*
ML: Muscle larvae
RNAi: RNA interference
siRNA: Small interfering RNA
TsGLS: *Trichinella spiralis* glutaminase
PCR: Polymerase chain reaction
AR: Acid resistance
qRT-PCR: Quantitative real-time PCR
IFA: Immunofluorescence assay
GAPDH: Glyceraldehyde-3-phosphate dehydrogenase
Gln: Glutamine
GLS: Glutaminase
