# Supplementary material for: RNAi-mediated silencing of Trichinella spiralis glutaminase results in reduced muscle larval infectivity
Source: Vet Res. 2021 Mar 25;52:51. doi: 10.1186/s13567-021-00921-1 (PMC7992778; doi:10.1186/s13567-021-00921-1)
Supplement: Supplementary file 1 — Additional file 1. siRNA sequences for TsGLS used in this study. [file 13567_2021_921_MOESM1_ESM.doc]

**Additional file 1** **Small interfering RNA (siRNA) sequences for *Trichinella spiralis* glutaminase (TsGLS) used in this study**

| **siRNA oligo** | **Sequence (5′-3′)** |
| --- | --- |
| siRNA-419 | sense：GCGACUGUACACUUGGAAAUG  anti-sense：CAUUUCCAAGUGUACAGUCGC |
| siRNA-881 | sense：GCUUUCCAGCUGGCGUAAAUU  anti-sense：AAUUUACGCCAGCUGGAAAGC |
| siRNA-1429 | sense：GCCGAUUACGAUGGUCGUACU  anti-sense：AGUACGACCAUCGUAAUCGGC |
| Control siRNA | sense：UUCUCCGAACGUGUCACGUTT  anti-sense：ACGUGACACGUUCGGAGAATT |
