# Supplementary figures and images for: RNAi-mediated silencing of Trichinella spiralis glutaminase results in reduced muscle larval infectivity
Source: Vet Res. 2021 Mar 25;52:51. doi: 10.1186/s13567-021-00921-1 (PMC7992778; doi:10.1186/s13567-021-00921-1)

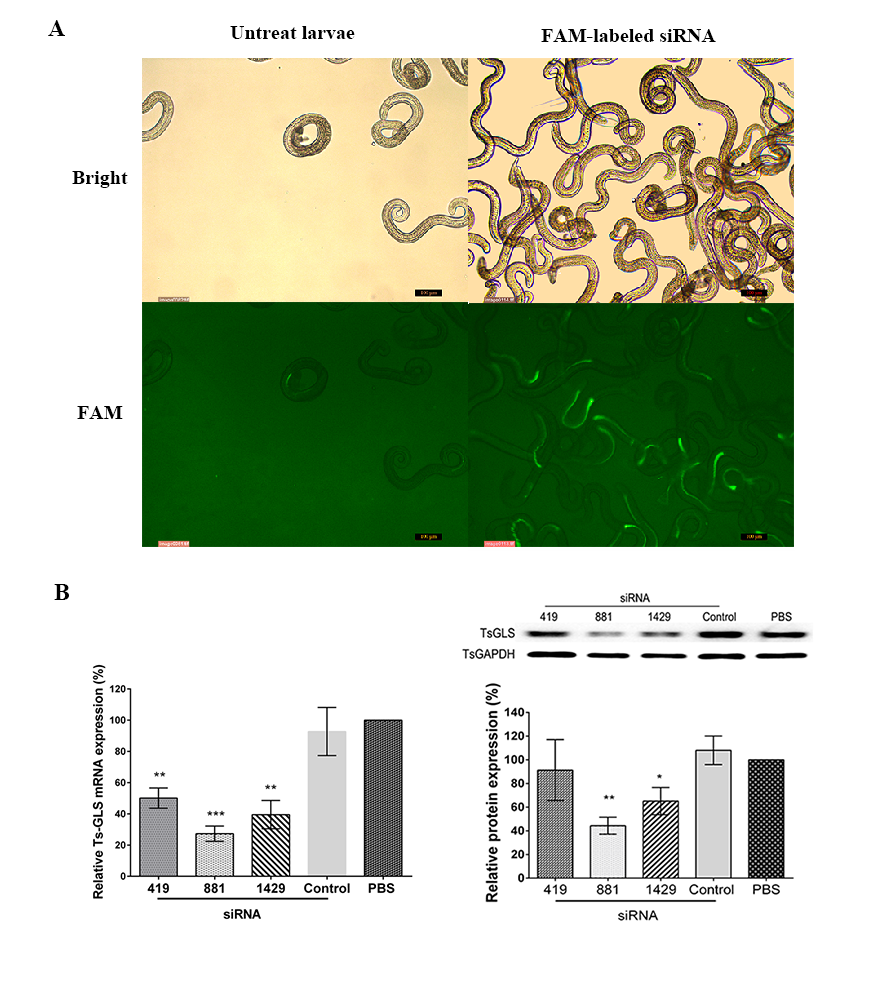

Supplement: Supplementary file 2 — Additional file 2. FAM-labeled control siRNA delivery to Trichinella spiralis ML. A: uptake of FAM-labeled siRNA into larvae at 12 h after soaking under a fluorescent microscope. No fluorescence was observed in the untreated larvae; B: relative transcription and expression levels of TsGLS mRNA and protein in T. spiralis ML 3 days after being soaked with different siRNAs. Western blot with specific antibodies showing the specific inhibition of TsGLS protein expression in extracts of T. spiralis larvae induced by siRNAs. Statistically significant differences with P < 0.05, P < 0.01 and P < 0.001 are indicated by *, **, and ***, respectively. [file 13567_2021_921_MOESM2_ESM.tif]

**A**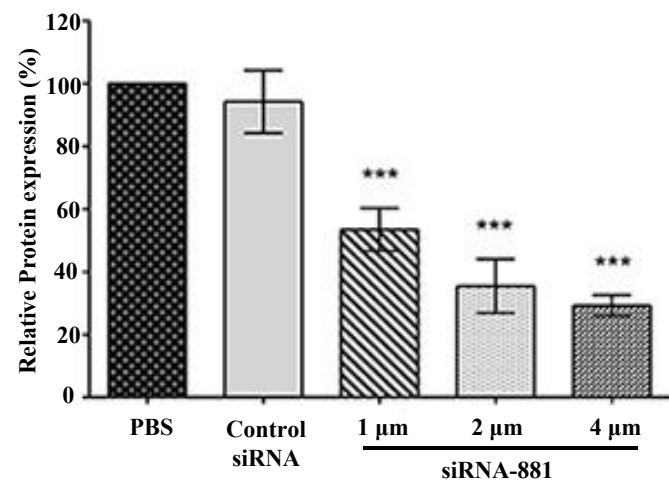**B**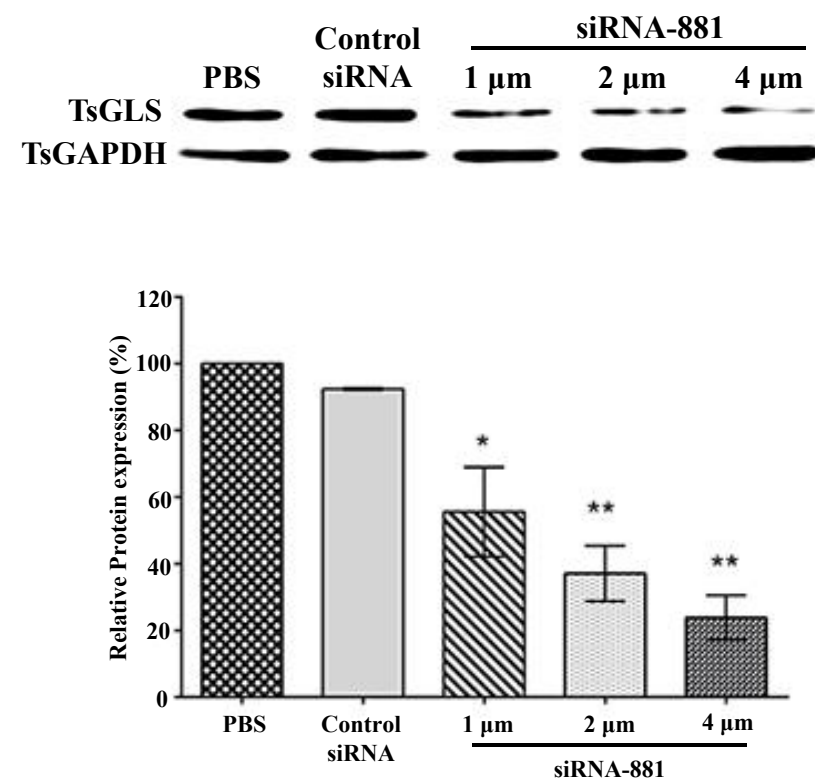**C**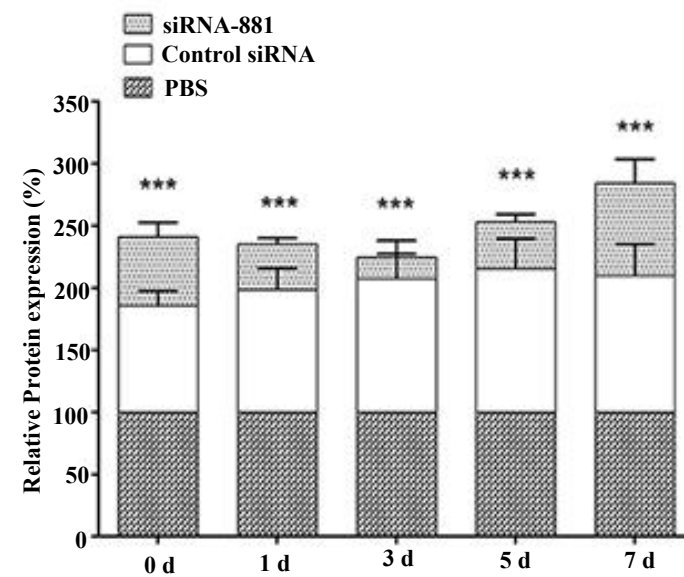

Supplement: Supplementary file 3 — Additional file 3. Optimization of experimental conditions for siRNA interference. A: relative transcription levels of TsGLS in larvae 3 days after being soaked with different concentrations of siRNA-881; B: expression levels of the TsGLS protein in larvae 3 days after being soaked with different concentrations of siRNA-881; C: relative transcription levels of TsGLS in larvae at 1, 3, 5, and 7 days after being electroporated with 2 μM of siRNA-881. All the assays were performed in triplicates, and statistically significant differences with P < 0.05 and P < 0.001 are indicated by * and ***, respectively. [file 13567_2021_921_MOESM3_ESM.pdf]

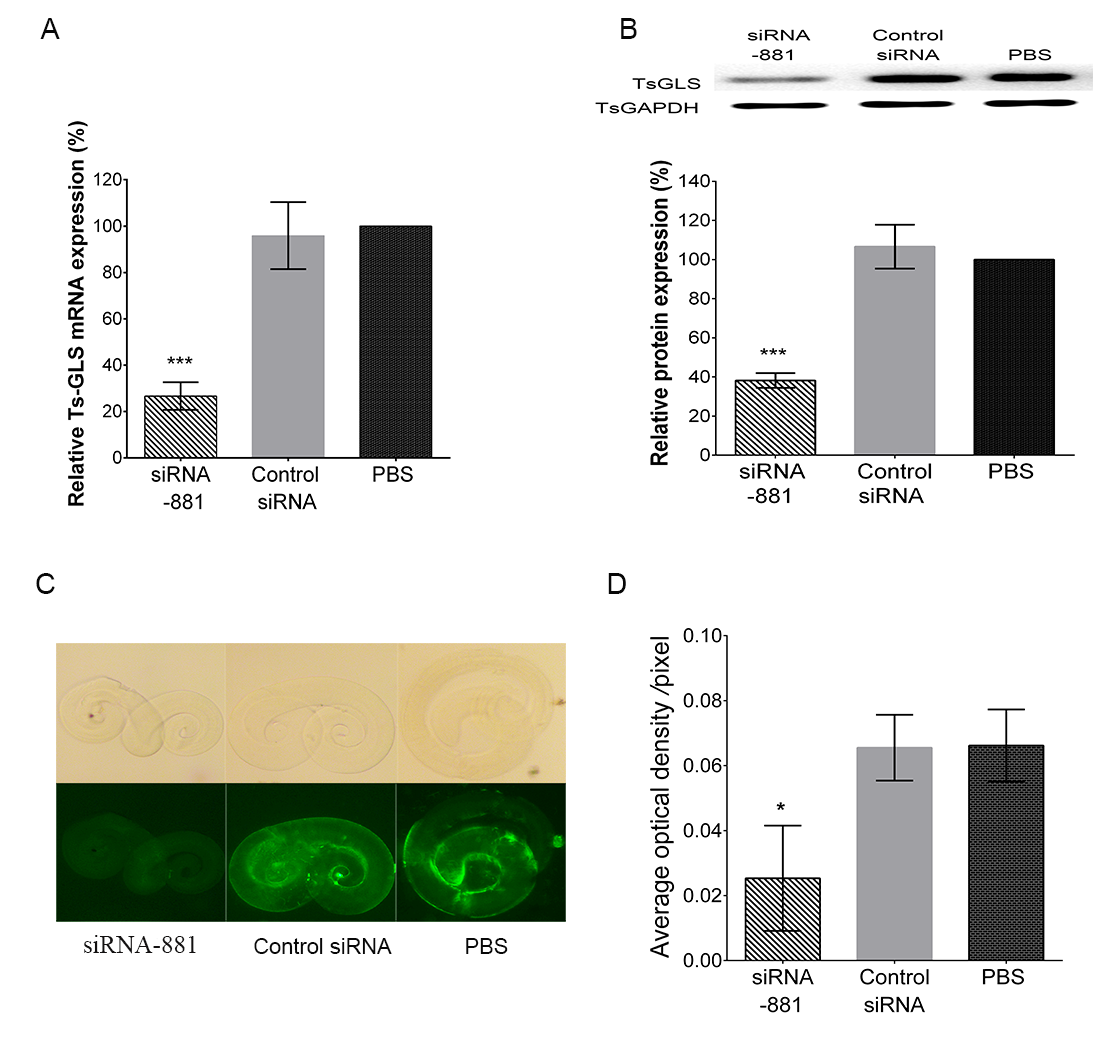

Supplement: Supplementary file 5 — Additional file 5. TsGLS effect on larvae after silencing the TsGLS gene. A: relative transcription levels of TsGLS in larvae after being soaked with siRNA-881; B: expression levels of the TsGLS protein in larvae after being soaked with siRNA-881; C: immunofluorescence signal analysis and average optical density after being soaked with siRNA-881. *: P < 0.05; **: P < 0.01; **: P < 0.001. [file 13567_2021_921_MOESM5_ESM.tif]

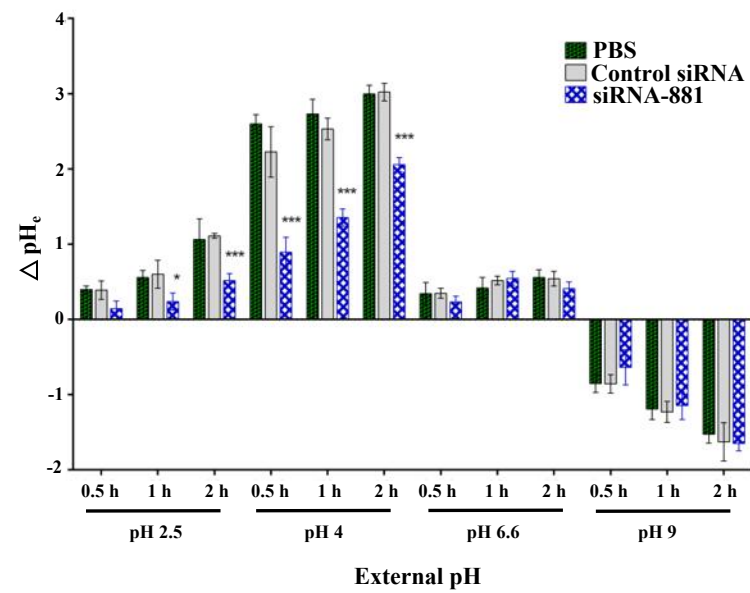

Supplement: Supplementary file 6 — Additional file 6. RNAi effect on the culture medium of siRNA-881-treated muscle larvae under different pH values and culture times. [file 13567_2021_921_MOESM6_ESM.pdf]
