# Supplementary material for: RNAi-mediated silencing of Trichinella spiralis glutaminase results in reduced muscle larval infectivity
Source: Vet Res. 2021 Mar 25;52:51. doi: 10.1186/s13567-021-00921-1 (PMC7992778; doi:10.1186/s13567-021-00921-1)
Supplement: Supplementary file 4 — Additional file 4. Survival rate after siRNA treatment of ML. [file 13567_2021_921_MOESM4_ESM.doc]

**Additional file 4 Survival rate after small interfering RNA (siRNA) treatment of muscle larvae (ML)**

| **Group** | **Survival number** | **Survival rate/%** |
| --- | --- | --- |
| siRNA-881 | 1280±110.6 | 64 |
| Control siRNA | 1247±102.7 | 62.35 |
| PBS | 1257±53.95 | 62.85 |

All assays were performed in quintuplicate, and data are presented as the mean ± SE
